# 1,5-Diamino­tetra­zolium chloride

**DOI:** 10.1107/S1600536810009633

**Published:** 2010-03-31

**Authors:** Ling-Qiao Meng, Zhi-Ming Du, Chun-Lin He, Xiao-Min Cong, Shuai Yang, Lin-Shuang Zhao

**Affiliations:** a State Key Laboratory of Explosion Science and Technology, Beijing Institute of Technology, Beijing 100081, People’s Republic of China

## Abstract

The title compound, CH_5_N_6_^+^·Cl^−^, crystallized with two indepedent 1,5-diamino­tetra­zolium cations and two independent chloride anions in the asymmetric unit. In the crystal, there are a number of N—H⋯Cl hydrogen-bonding inter­actions, which generate a three-dimensional network.

## Related literature

For the preparation of the starting material, 1,5-diamino­tetra­zole, see: Galvez-Ruiz *et al.* (2005 ▶). For the preparation of 5-amino­tetra­zolium halogenide salts, see: Denffer *et al.* (2008 ▶) and of 1,5-diamino­tetra­zolium hydro­chloride, see: He *et al.* (2009*a* ▶). For the bond distances and angles in a related structure, see: He *et al.* (2009*b* ▶). For van der Waals radii, see: http://biblo.chm.uri.edu/PeriodicTable/PeriodicTableoftheElements.htm. 
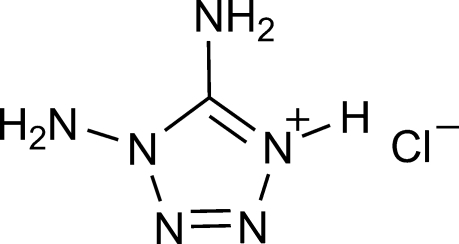


## Experimental

### 

#### Crystal data

CH_5_N_6_^+^·Cl^−^
*M* *_r_* = 136.56
Orthorhombic, 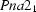

*a* = 12.389 (3) Å
*b* = 6.4500 (12) Å
*c* = 13.305 (3) Å
*V* = 1063.1 (4) Å^3^
*Z* = 8
Mo *K*α radiation
μ = 0.61 mm^−1^
*T* = 93 K
0.43 × 0.27 × 0.10 mm

#### Data collection

Rigaku AFC10/Saturn724+ diffractometer
Absorption correction: multi-scan (*CrystalClear*; Rigaku, 2008 ▶) *T*_min_ = 0.778, *T*_max_ = 0.942
7927 measured reflections
1268 independent reflections
1246 reflections with *I* > 2σ(*I*)
*R*_int_ = 0.029

#### Refinement

*R*[*F* ^2^ > 2σ(*F* ^2^)] = 0.025
*wR*(*F* ^2^) = 0.061
*S* = 1.07
1268 reflections
185 parameters
1 restraint
All H-atom parameters refined
Δρ_max_ = 0.64 e Å^−3^
Δρ_min_ = −0.17 e Å^−3^

### 

Data collection: *CrystalClear* (Rigaku, 2008 ▶); cell refinement: *CrystalClear*; data reduction: *CrystalClear*; program(s) used to solve structure: *SHELXS97* (Sheldrick, 2008 ▶); program(s) used to refine structure: *SHELXL97* (Sheldrick, 2008 ▶); molecular graphics: *PLATON* (Spek, 2009 ▶); software used to prepare material for publication: *WinGX* (Farrugia, 1999 ▶).

## Supplementary Material

Crystal structure: contains datablocks I, global. DOI: 10.1107/S1600536810009633/su2159sup1.cif

Structure factors: contains datablocks I. DOI: 10.1107/S1600536810009633/su2159Isup2.hkl

Additional supplementary materials:  crystallographic information; 3D view; checkCIF report

## Figures and Tables

**Table 1:** Hydrogen-bond geometry (Å, °)

*D*—H⋯*A*	*D*—H	H⋯*A*	*D*⋯*A*	*D*—H⋯*A*
N4—H4⋯Cl2	0.93 (4)	2.16 (4)	3.017 (2)	154 (4)
N5—H5*A*⋯Cl1^i^	0.79 (4)	2.77 (4)	3.555 (3)	179 (6)
N5—H5*B*⋯Cl2^ii^	0.94 (4)	2.39 (4)	3.317 (2)	170 (3)
N6—H6*A*⋯Cl1^iii^	0.86 (4)	2.65 (4)	3.376 (3)	142 (3)
N6—H6*B*⋯Cl2^iv^	0.93 (4)	2.25 (4)	3.146 (2)	162 (3)
N10—H10⋯Cl1^i^	0.79 (4)	2.30 (4)	3.021 (2)	152 (4)
N11—H11*A*⋯Cl2^v^	0.96 (4)	2.65 (4)	3.567 (3)	161 (3)
N11—H11*B*⋯Cl1	0.85 (4)	2.47 (4)	3.306 (2)	170 (3)
N12—H12*A*⋯Cl2^vi^	0.81 (4)	2.67 (4)	3.388 (3)	148 (3)
N12—H12*B*⋯Cl1^vii^	0.80 (4)	2.39 (4)	3.173 (2)	171 (4)

Symmetry codes: (i) 
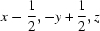
; (ii) 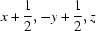; (iii) 
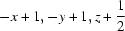
; (iv) 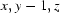; (v) 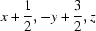; (vi) 
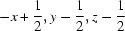
; (vii) 
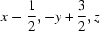
.

**Fig. 1.**
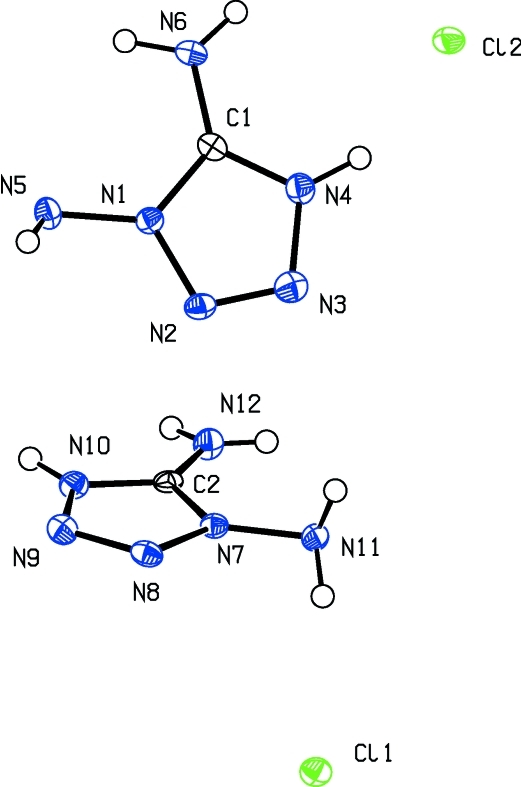
The molecular structure of the title compound with the atom-numbering scheme. Displacement ellipsoids are drawn at the 50% probability level.

## supplementary crystallographic information

### Comment

The synthesis and study of nitrogen-rich energetic salts and highly energetic materials for possible military as well as civil applications has attracted considerable interest in recent years, especially the salts with tetrazole-containing compounds (Galvez-Ruiz *et al.*, 2005; Denffer *et al.*, 2008). The nitrogen content of 5-amniotetrazole and 1,5-diaminotetrazole, which are primary sources for preparing energetic salts, is 82.3% and 84%, respectively. Denffer *et al.* (2008) reported the synthesis of 5-aminotetrazolium hydrochloride and determinated its crystal structure. Our rearch group has recently reported on the synthesis of the title compound (He *et al.*., 2009a,b), and herein we report on its crystal structure.

The molecular structure of the title molecule is presented in Fig. 1. It crystallizes with two independent 1,5-Diaminotetrazolium cations and two independent chloride anions per asymmetric unit. The bond distances and angles are as expected for a molecule of this kind, and are similar to the corresponding distances and angles reported by (He *et al.*, 2009a,b). The cations, excluding the N6 and N11 hydrogen atoms, are planar (maximum deviation 0.020 (2) Å).

The distance between the Cl1 anion and the plane formed by the cation ring 1, (= N1,N2,N3,N4,C1), is 0.445 (1) Å, and the perpendicular distances of this cation centroid, Cg1, to the parallel cation 2 ring planes (= N7,N8,N9,N10,C2), are 2.868 (1) Å (symmetry code: 1-x, -y, 0.5+z) and 2.922 (1) Å (symmetry code: 1-x, 1-y, 0.5+z). The distances of N2—C2 (2.864 (4) Å) and N8—C1^i^ (2.883 (4) Å) [symmetry code (i) = 0.5+x, 0.5-y, z] are smaller than the sum of the associated van der Waals Radii (*r*_N_ + *r*_C_ = 3.25 Å), because of edge-to-face π-π interactions between the two cations. Both of the amino groups, in position 4 (N4) and position 5 (N6), form a long contact to the Cl2^-^ anion (N4—Cl2 = 3.017 (2) Å and N6—Cl2^ii^ = 3.146 (2) Å [symmetry code (ii) = x, 1+y, z]), which is within the sum of the van der Waals radii (*r*_N_ +*r*_Cl_ = 3.30 Å).

In the crystal there are a number of N-H···Cl hydrogen bonds which result in the foamation of a three-dimensional network (Table 1).

### Experimental

The starting material, 1,5-diaminotetrazole, was prepared according to the literature method (Galvez-Ruiz *et al.*, 2005). 1,5-diaminotetrazole (2.0043 g, 20.04 mmol) suspended in 40 mL of methanol, was reacted with 10 mL concentrated HCl. The reaction mixture was refluxed for 2 h and then the solvent was evaporated until precipitation occured. The concentrated solution was then placed in the refrigerator, and the white 1,5-diaminotetrazolium hydrochloride was obtained. The precipitate was filtered off and washed with water. The crude product was recrystallized from methanol (Yield: 2.4189 g, 88.6%). Crystals suitable for X-ray structure determination were obtained by slow evaporation of a solution in methanol at rt.

### Refinement

In the final cycles of refinement, in the absence of significant anomalous scattering effects, Friedel pairs were merged and Δf " set to zero. All the H-atoms were located in difference Fourier maps and were freely refined: N-H = 0.79 (4) - 0.96 (4) Å.

### Crystal data

**Table:**

CH_5_N_6_^+^·Cl^−^	*F*(000) = 560
*M**_r_* = 136.56	*D*_x_ = 1.706 Mg m^−^^3^
Orthorhombic, *P**n**a*2_1_	Mo *K*α radiation, λ = 0.71073 Å
Hall symbol: P 2c -2n	Cell parameters from 3581 reflections
*a* = 12.389 (3) Å	θ = 3.1–27.5°
*b* = 6.4500 (12) Å	µ = 0.61 mm^−^^1^
*c* = 13.305 (3) Å	*T* = 93 K
*V* = 1063.1 (4) Å^3^	Prism, colourless
*Z* = 8	0.43 × 0.27 × 0.10 mm

### Data collection

**Table:**

Rigaku AFC10/Saturn724+ diffractometer	1268 independent reflections
Radiation source: Rotating Anode	1246 reflections with *I* > 2σ(*I*)
graphite	*R*_int_ = 0.029
Detector resolution: 28.5714 pixels mm^-1^	θ_max_ = 27.5°, θ_min_ = 3.1°
Multi–scan	*h* = −16→14
Absorption correction: multi-scan (*CrystalClear*; Rigaku, 2008)	*k* = −8→8
*T*_min_ = 0.778, *T*_max_ = 0.942	*l* = −16→17
7927 measured reflections	

### Refinement

**Table:**

Refinement on *F*^2^	Primary atom site location: structure-invariant direct methods
Least-squares matrix: full	Secondary atom site location: difference Fourier map
*R*[*F*^2^ > 2σ(*F*^2^)] = 0.025	Hydrogen site location: difference Fourier map
*wR*(*F*^2^) = 0.061	All H-atom parameters refined
*S* = 1.07	*w* = 1/[σ^2^(*F*_o_^2^) + (0.0395*P*)^2^ + 0.2133*P*] where *P* = (*F*_o_^2^ + 2*F*_c_^2^)/3
1268 reflections	(Δ/σ)_max_ = 0.004
185 parameters	Δρ_max_ = 0.64 e Å^−^^3^
1 restraint	Δρ_min_ = −0.17 e Å^−^^3^

### Special details

**Table:**

Geometry. Bond distances, angles etc. have been calculated using the rounded fractional coordinates. All su's are estimated from the variances of the (full) variance-covariance matrix. The cell esds are taken into account in the estimation of distances, angles and torsion angles
Refinement. Refinement of F^2^ against ALL reflections. The weighted R-factor wR and goodness of fit S are based on F^2^, conventional R-factors R are based on F, with F set to zero for negative F^2^. The threshold expression of F^2^ > 2sigma(F^2^) is used only for calculating R-factors(gt) etc. and is not relevant to the choice of reflections for refinement. R-factors based on F^2^ are statistically about twice as large as those based on F, and R- factors based on ALL data will be even larger.

### Fractional atomic coordinates and isotropic or equivalent isotropic displacement parameters (Å^2^)

**Table:**

	*x*	*y*	*z*	*U*_iso_*/*U*_eq_	
N1	0.44089 (16)	0.1470 (3)	0.39856 (16)	0.0134 (6)	
N2	0.51729 (18)	0.2805 (3)	0.36265 (19)	0.0157 (6)	
N3	0.48306 (18)	0.4641 (3)	0.37753 (18)	0.0191 (7)	
N4	0.38470 (18)	0.4512 (3)	0.42323 (16)	0.0170 (6)	
N5	0.44793 (18)	−0.0680 (3)	0.39178 (19)	0.0174 (6)	
N6	0.26869 (18)	0.1788 (3)	0.4778 (2)	0.0173 (6)	
C1	0.3577 (2)	0.2537 (4)	0.4364 (2)	0.0129 (7)	
N7	0.68577 (16)	0.3860 (3)	0.20486 (15)	0.0128 (6)	
N8	0.76391 (17)	0.2503 (3)	0.23810 (19)	0.0154 (6)	
N9	0.73281 (18)	0.0683 (3)	0.21667 (17)	0.0170 (6)	
N10	0.63448 (17)	0.0824 (3)	0.17032 (17)	0.0153 (6)	
N11	0.68953 (18)	0.5995 (3)	0.21790 (18)	0.0154 (6)	
N12	0.51472 (17)	0.3555 (3)	0.1248 (2)	0.0167 (6)	
C2	0.6038 (2)	0.2794 (4)	0.1630 (2)	0.0131 (7)	
Cl1	0.93591 (4)	0.67650 (9)	0.13004 (5)	0.0168 (2)	
Cl2	0.19017 (4)	0.71516 (9)	0.47049 (5)	0.0174 (2)	
H4	0.342 (3)	0.565 (7)	0.438 (3)	0.048 (12)*	
H5A	0.446 (3)	−0.093 (7)	0.334 (3)	0.037 (11)*	
H5B	0.514 (3)	−0.104 (5)	0.422 (3)	0.031 (9)*	
H6A	0.215 (3)	0.259 (6)	0.492 (3)	0.037 (10)*	
H6B	0.260 (3)	0.037 (6)	0.469 (3)	0.040 (10)*	
H10	0.599 (3)	−0.016 (6)	0.158 (3)	0.028 (9)*	
H11A	0.677 (3)	0.623 (5)	0.288 (3)	0.024 (8)*	
H11B	0.753 (3)	0.634 (5)	0.200 (2)	0.016 (7)*	
H12A	0.477 (3)	0.275 (5)	0.094 (3)	0.021 (9)*	
H12B	0.502 (3)	0.476 (6)	0.129 (3)	0.028 (9)*	

### Atomic displacement parameters (Å^2^)

**Table:**

	*U*^11^	*U*^22^	*U*^33^	*U*^12^	*U*^13^	*U*^23^
N1	0.0118 (10)	0.0145 (10)	0.0140 (10)	−0.0003 (8)	−0.0009 (8)	0.0006 (8)
N2	0.0157 (11)	0.0187 (10)	0.0126 (12)	−0.0025 (8)	0.0014 (9)	0.0005 (8)
N3	0.0194 (11)	0.0213 (12)	0.0166 (12)	−0.0018 (8)	−0.0005 (10)	−0.0002 (10)
N4	0.0165 (11)	0.0161 (10)	0.0184 (11)	0.0005 (8)	−0.0008 (9)	−0.0015 (9)
N5	0.0182 (11)	0.0141 (10)	0.0198 (12)	0.0029 (8)	0.0002 (9)	−0.0009 (9)
N6	0.0132 (9)	0.0197 (11)	0.0189 (11)	0.0007 (8)	0.0042 (10)	−0.0004 (10)
C1	0.0118 (12)	0.0175 (11)	0.0093 (12)	0.0023 (9)	−0.0028 (9)	−0.0006 (9)
N7	0.0112 (9)	0.0146 (10)	0.0125 (10)	0.0000 (8)	−0.0001 (8)	0.0008 (8)
N8	0.0115 (11)	0.0202 (10)	0.0144 (12)	0.0026 (8)	0.0003 (9)	0.0007 (8)
N9	0.0170 (11)	0.0184 (11)	0.0156 (11)	0.0014 (8)	−0.0002 (9)	−0.0001 (9)
N10	0.0124 (10)	0.0164 (10)	0.0171 (10)	−0.0023 (8)	−0.0004 (8)	−0.0027 (9)
N11	0.0126 (10)	0.0137 (10)	0.0199 (12)	−0.0020 (8)	0.0008 (9)	−0.0013 (9)
N12	0.0134 (9)	0.0170 (11)	0.0196 (11)	−0.0020 (8)	−0.0037 (10)	−0.0016 (11)
C2	0.0134 (12)	0.0165 (12)	0.0094 (12)	−0.0031 (9)	0.0028 (10)	−0.0010 (9)
Cl1	0.0114 (3)	0.0194 (3)	0.0197 (3)	−0.0004 (2)	−0.0017 (3)	0.0002 (3)
Cl2	0.0126 (3)	0.0196 (3)	0.0199 (3)	−0.0011 (2)	0.0019 (3)	−0.0003 (3)

### Geometric parameters (Å, °)

**Table:**

N1—N2	1.366 (3)	N7—N8	1.378 (3)
N1—N5	1.392 (3)	N7—N11	1.389 (3)
N1—C1	1.338 (3)	N7—C2	1.347 (3)
N2—N3	1.273 (3)	N8—N9	1.268 (3)
N3—N4	1.364 (3)	N9—N10	1.368 (3)
N4—C1	1.329 (3)	N10—C2	1.330 (3)
N6—C1	1.324 (3)	N12—C2	1.310 (3)
N4—H4	0.93 (4)	N10—H10	0.79 (4)
N5—H5A	0.79 (4)	N11—H11A	0.96 (4)
N5—H5B	0.94 (4)	N11—H11B	0.85 (4)
N6—H6A	0.86 (4)	N12—H12A	0.81 (4)
N6—H6B	0.93 (4)	N12—H12B	0.80 (4)
			
N2—N1—N5	124.2 (2)	N7—N8—N9	107.6 (2)
N2—N1—C1	109.95 (19)	N8—N9—N10	108.08 (19)
N5—N1—C1	125.8 (2)	N9—N10—C2	110.6 (2)
N1—N2—N3	107.5 (2)	C2—N10—H10	126 (3)
N2—N3—N4	108.06 (19)	N9—N10—H10	122 (3)
N3—N4—C1	110.0 (2)	N7—N11—H11B	105 (2)
N3—N4—H4	124 (2)	N7—N11—H11A	105.9 (19)
C1—N4—H4	126 (3)	H11A—N11—H11B	112 (3)
N1—N5—H5A	105 (3)	C2—N12—H12B	120 (3)
H5A—N5—H5B	113 (4)	C2—N12—H12A	116 (3)
N1—N5—H5B	106 (2)	H12A—N12—H12B	123 (4)
C1—N6—H6A	121 (3)	N4—C1—N6	127.9 (2)
C1—N6—H6B	114 (2)	N1—C1—N4	104.5 (2)
H6A—N6—H6B	122 (3)	N1—C1—N6	127.6 (2)
N8—N7—N11	124.48 (19)	N7—C2—N12	127.2 (2)
N8—N7—C2	109.76 (19)	N10—C2—N12	128.8 (2)
N11—N7—C2	125.7 (2)	N7—C2—N10	104.0 (2)
			
N5—N1—N2—N3	177.3 (2)	N11—N7—N8—N9	177.8 (2)
C1—N1—N2—N3	0.0 (3)	C2—N7—N8—N9	0.9 (3)
N2—N1—C1—N4	−0.2 (3)	N8—N7—C2—N10	−0.9 (3)
N2—N1—C1—N6	180.0 (3)	N8—N7—C2—N12	178.8 (3)
N5—N1—C1—N4	−177.4 (2)	N11—N7—C2—N10	−177.7 (2)
N5—N1—C1—N6	2.8 (4)	N11—N7—C2—N12	1.9 (4)
N1—N2—N3—N4	0.1 (3)	N7—N8—N9—N10	−0.5 (3)
N2—N3—N4—C1	−0.2 (3)	N8—N9—N10—C2	−0.1 (3)
N3—N4—C1—N1	0.2 (3)	N9—N10—C2—N7	0.6 (3)
N3—N4—C1—N6	−179.9 (3)	N9—N10—C2—N12	−179.0 (3)

### Hydrogen-bond geometry (Å, °)

**Table:**

*D*—H···*A*	*D*—H	H···*A*	*D*···*A*	*D*—H···*A*
N4—H4···Cl2	0.93 (4)	2.16 (4)	3.017 (2)	154 (4)
N5—H5A···Cl1^i^	0.79 (4)	2.77 (4)	3.555 (3)	179 (6)
N5—H5B···Cl2^ii^	0.94 (4)	2.39 (4)	3.317 (2)	170 (3)
N6—H6A···Cl1^iii^	0.86 (4)	2.65 (4)	3.376 (3)	142 (3)
N6—H6B···Cl2^iv^	0.93 (4)	2.25 (4)	3.146 (2)	162 (3)
N10—H10···Cl1^i^	0.79 (4)	2.30 (4)	3.021 (2)	152 (4)
N11—H11A···Cl2^v^	0.96 (4)	2.65 (4)	3.567 (3)	161 (3)
N11—H11B···Cl1	0.85 (4)	2.47 (4)	3.306 (2)	170 (3)
N12—H12A···Cl2^vi^	0.81 (4)	2.67 (4)	3.388 (3)	148 (3)
N12—H12B···Cl1^vii^	0.80 (4)	2.39 (4)	3.173 (2)	171 (4)

Symmetry codes: (i) *x*−1/2, −*y*+1/2, *z*; (ii) *x*+1/2, −*y*+1/2, *z*; (iii) −*x*+1, −*y*+1, *z*+1/2; (iv) *x*, *y*−1, *z*; (v) *x*+1/2, −*y*+3/2, *z*; (vi) −*x*+1/2, *y*−1/2, *z*−1/2; (vii) *x*−1/2, −*y*+3/2, *z*.

## References

[bb1] Denffer, V. M., Klapötke, T. M. & Sabaté, C. M. (2008). *Z. Anorg. Allg. Chem.* **634**, 2575–2582.

[bb2] Farrugia, L. J. (1999). *J. Appl. Cryst.* **32**, 837–838.

[bb3] Galvez-Ruiz, J. C., Holl, G., Karaghiosoff, K., Klapötke, T. M., Lohnwitz, K., Mayer, P., Noth, H., Polborn, K., Rohbogner, C. J., Suter, M. & Weigand, J. J. (2005). *Inorg. Chem.* **44**, 4237–4253. 10.1021/ic050104g 15934752

[bb4] He, C. L., Du, Z. M., Cong, X. M., Tang, Z. Q. & Meng, L. Q. (2009*a*). *Theory and Practice of Energetic Materials*, Vol. 8, pp. 673–677. Beijing Institute of Technology.

[bb5] He, C.-L., Du, Z.-M., Tang, Z.-Q., Cong, X.-M. & Meng, L.-Q. (2009*b*). *Acta Cryst.* E**65**, o1760. 10.1107/S1600536809024994 PMC2977296 21583470

[bb6] Rigaku (2008). *CrystalClear* Rigaku Corporation, Tokyo, Japan.

[bb7] Sheldrick, G. M. (2008). *Acta Cryst.* A**64**, 112–122. 10.1107/S0108767307043930 18156677

[bb8] Spek, A. L. (2009). *Acta Cryst.* D**65**, 148–155. 10.1107/S090744490804362X PMC2631630 19171970

